# GPU-Based 3D Cone-Beam CT Image Reconstruction for Large Data Volume

**DOI:** 10.1155/2009/149079

**Published:** 2009-08-26

**Authors:** Xing Zhao, Jing-jing Hu, Peng Zhang

**Affiliations:** ^1^School of Mathematical Sciences, Capital Normal University, Beijing 100048, China; ^2^Department of Computer Science, Beijing Institute of Technology, Beijing 100081, China

## Abstract

Currently, 3D cone-beam CT image reconstruction speed is still a severe limitation for clinical application. The computational power of modern graphics processing units (GPUs) has been harnessed to provide impressive acceleration of 3D volume image reconstruction. For extra large data volume exceeding the physical graphic memory of GPU, a straightforward compromise is to divide data volume into blocks. Different from the conventional Octree partition method, a new partition scheme is proposed in this paper. This method divides both projection data and reconstructed image volume into subsets according to geometric symmetries in circular cone-beam projection layout, and a fast reconstruction for large data volume can be implemented by packing the subsets of projection data into the RGBA channels of GPU, performing the reconstruction chunk by chunk and combining the individual results in the end. The method is evaluated by reconstructing 3D images from computer-simulation data and real micro-CT data. Our results indicate that the GPU implementation can maintain original precision and speed up the reconstruction process by 110–120 times for circular cone-beam scan, as compared to traditional CPU implementation.

## 1. Introduction

Computed Tomography (CT) has become one of the most popular diagnostic modalities since its invention thirty years ago. Compared with 2D parallel-beam and fan-beam CT, 3D cone-beam CT system is able to achieve higher special resolution and better utilization of photons [1]. With the rapid development of detector technology, the single detector unit is getting smaller and smaller while the number of detector units is becoming larger and larger. This means that there will be larger amount of projection data needed to be processed in 3D cone-beam CT system. For example, PaxScan2520, a flat panel detector made by Varian, has 1920 × 1536 detector units. The output of each detector unit is 16 bits. The size of projection data is about 4 GB for a 720-view CT scan. The size of reconstruction image is about 512 MB for a 512^3^ image array and is 4 GB for a 1024^3^ image array. The gigabyte data size is huge even for a graphic workstation. Currently, image reconstruction speed is still a bottleneck for the development of 3D cone-beam CT. The study of fast and efficient reconstruction algorithms for large volume image and their implementation on hardware or software will have important significance both theoretically and practically [2, 3]. 

 The Graphics Processing Unit (GPU) can process volume data in parallel when working in single instruction multiple data (SIMD) mode [4]. Because of the increasing demand of computer game market and engineering design, the development of GPU has been much faster than CPU. Nowadays the processing capability of GPU is increasing dramatically. The increasing programmability of GPU has made it possible that certain general purpose computing based on CPU can be implemented on GPU with a much faster computation speed, and general purpose GPU computing has become another hot research topic, which includes its application on CT image reconstruction [3, 5]. 

 Back to 1990s, only high-end workstations, such as the SGI Octane or Onyx, had the level of graphics hardware necessary for CT image reconstruction. Cabral et al. were the first to employ this hardware for the acceleration of CT reconstruction [6]. With the fast development of the low-cost PC-based graphics hardware of similar capabilities than that of the SGI, many researchers recently have tried to implement both iterative and analytic CT image reconstruction using GPU-acceleration [5, 7–10]. Usually the size of graphics card memory is much less than system memory, which imposes a big constraint on the GPU-based image reconstruction. The size of the volume image array for most GPU-accelerated CT reconstruction is generally limited to 512^3^. Muller and Xu have studied the CT image reconstruction for large volume data [8]. They divided the target volume using a method similar to Octree and proposed to reconstruction those small bricks one by one for large volume data. Schiwietz et al. also presented a memory management strategy that decreases the bus transfer between main memory and GPU memory for reconstructing large volume [9]. In this paper, we study how to partition the data to fit them into the graphics card memory. A new method of the projection data partition for large volume data is proposed. According to rotational symmetry and vertical symmetry in circular cone-beam projection layout, the method divides both projection data and reconstructed image volume into subsets. By packing the subsets of data into the RGBA channels of GPU, a fast reconstruction for large data volume can be implemented. 

 This paper is organized as follows. In Section 2, our GPU-accelerated backward-projection for FDK algorithm is introduced, and then the utilization of geometric symmetries is described. In Section 3, the partition scheme for reconstructing large data volume is presented. In Section 4, numerical experiments on various datasets are presented to evaluate the speedups with our method. In Section 5, relevant issues are discussed.

## 2. Methods

The filtered back-projection algorithm proposed by Feldkamp, Davis and Kress (FDK) for 3D volume reconstruction from circular cone-beam projections still remains one of the most widely used approach [11]. In this algorithm, the most time-consuming part is the back-projection procedure, which has a complexity of *O*(*N* ^4^) in the spatial domain and constitutes the bottleneck for all software solutions [8]. So here we purposely concentrate on using GPU to accelerate the backward-projection of the reconstruction program. Because the backward-projection is very similar for different CT image reconstruction algorithms, it will be easy to adapt our scheme into different reconstruction algorithms.

### 2.1. GPU Accelerated Backward-Projection for FDK Algorithm

**Figure 1:**
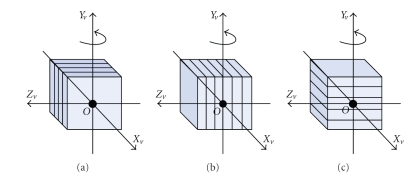
Axis-aligned stack of 2D-textured slices for representing reconstructed volume. (a) along *X*_*v*_ axis aligned stack, (b) along *Z_v_* axis aligned stack, and (c) along the rotation axis *Y*_*v*_ aligned stack.

Current GPUs can be used either as a graphical pipeline or as a multiprocessor chip thanks to the CUDA interface from Nvidia. For both options, the acceleration factor of GPU is high. Xu and Mueller have observed that an implementation of the cone beam back-projection using the graphics pipeline is 3 times faster than the one made with CUDA interface [12]. Hence we use the graphics pipeline to accelerate CT reconstruction in this paper. In order to harness GPU to provide acceleration of 3D volume image reconstruction, we represent reconstructed volume as an axis-aligned stack of 2D-textured slices. The volume may be represented by three kinds of proxy geometries as shown in Figure 1. If the stack of 2D-textured slices aligned along the rotation axis *Y*_*v*_ (Figure 1(c)) is adopted, only one data set is enough for circular cone-beam reconstruction. Otherwise, two copies of the data set should be used simultaneously in GPU memory for decreasing the inconsistent sampling rate of volume. This can cause bottlenecks when the memory bandwidth is less than the compute bandwidth, and also needs to merge the two textured slices stacks in each backward-projection loop [13]. Hence, we choose the model shown in Figure 1(c) as reconstructed volume model.

**Figure 2:**
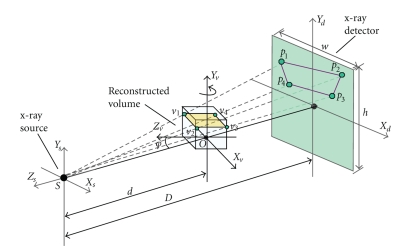
Geometry of back projection. The slice under reconstruction has each filtered x-ray image projected onto it by projective texture mapping.

Figure 2 shows the geometry between the X-ray source, the reconstructed volume and the detector of cone-beam CT. The source-to-rotation center distance is *d*, the source-to-detector distance is *D*, and the rotation axis of reconstructed volume is *Y*_*v*_. The target volume is represented as a stack of 2D slices (textures) perpendicularly aligned along *Y*_*v*_ axis. The key for the GPU-accelerated backward-projection is to calculate the projection positions of the vertices of every slice on the X-ray detector of cone-beam CT system. If the projection positions of the four vertices of a slice on the X-ray detector are produced, we can generate the projection coordinate of each voxel of the slice by interpolating the coordinates of the vertices in GPU rasterizer, and achieve the backward-projection from a projection image to the slice in GPU fragment shader. 

 Here is our detailed algorithm for backward-projecting a projection image to a volume slice based on GPU. As shown in Figure 2, the slice under reconstruction has four vertices *v*_1_, *v*_2_, *v*_3_, and *v*_4_, whose projection positions in detector space are *p*_1_, *p*_2_, *p*_3_, and *p*_4_, respectively. According to projective-texture mapping theory [14, 15], we decompose and express the full coordinate transformation from volume space to detector space as a series of matrices, as shown in formula (1). As compared with the method presented in [12], formula (1) focuses on calculating the projection coordinates of any one vertex of a slice in circular cone-beam projection layout. The coordinates in detector space of the four projection positions may be computed in parallel in GPU according to this formula:

(1) $$\begin{array}{c} E\times P\times T\times R\times v=v_{p},\\ \left[\begin{array}{cccc} \frac{N_{w}}{2} & 0 & 0 & \frac{N_{w}}{2}\\ 0 & \frac{N_{h}}{2} & 0 & \frac{N_{h}}{2}\\ 0 & 0 & 1 & 1\\ 0 & 0 & 0 & 1 \end{array}\right]\left[\begin{array}{cccc} \frac{2D}{w} & 0 & 0 & 0\\ 0 & \frac{2D}{h} & 0 & 0\\ 0 & 0 & \frac{-\left(f+n\right)}{f-n} & \frac{-2fn}{f-n}\\ 0 & 0 & -1 & 0 \end{array}\right]\times \\ \left[\begin{array}{cccc} 1 & 0 & 0 & 0\\ 0 & 1 & 0 & 0\\ 0 & 0 & 1 & -d\\ 0 & 0 & 0 & 1 \end{array}\right]\left[\begin{array}{cccc} \cos\;\varphi & 0 & -\sin\varphi & 0\\ 0 & 1 & 0 & 0\\ \sin\varphi & 0 & \cos\;\varphi & 0\\ 0 & 0 & 0 & 1 \end{array}\right]\left[\begin{array}{c} x_{v}\\ y_{v}\\ z_{v}\\ 1 \end{array}\right]=\left[\begin{array}{c} x_{p}\\ y_{p}\\ z_{p}\\ w_{p} \end{array}\right]\begin{array}{c} . \end{array} \end{array}$$

In formula (1), the coordinate of a slice vertex in volume coordinate system is expressed as a 4D homogenous vector **v** = (*x*_*v*_, *y*_*v*_, *z*_*v*_, 1)^*T*^. A 4 × 4 rotation matrix **R** rotates the volume coordinate system by *φ* degrees in counter-clockwise direction. Another 4 × 4 translation matrix **T** translates the volume coordinate system a distance of *d* along the negative axis. The two matrices **R** and **T** jointly map the vertex coordinate **v** from volume coordinate system (*X*_*v*_ − *Y*_*v*_ − *Z*_*v*_) into source coordinate system (*X*_*s*_ − *Y*_*s*_ − *Z*_*s*_). A 4 × 4 perspective projection matrix **P**, determined by the source location and the detector dimensions *w* and *h*, defines a frustum for cone-beam projection. The parameters *n* and *f* of the matrix **P** denote the distances from x-ray source to the near and far clipping planes of the frustum, respectively. The matrix **P** implements the subsequent perspective projection that maps the frustum into a cube clip space, whose Cartesian coordinates are between −1 and 1. Then a texture coordinate conversion matrix **E**, defined by the horizontal and vertical numbers of detector units, produces the homogeneous coordinates **v**_*p*_ in detector space for the vertex **v**. 

 By implementing the calculation of formula (1) in SIMD way in the vertex shader of GPU, the rectangle texture homogeneous coordinates of the four projection positions *p*_1_, *p*_2_, *p*_3_, and *p*_4_ in detector space are obtained. Then the fragments corresponding to the voxels of slice are generated in orthographic viewing mode in GPU rasterizer, and the texture homogeneous coordinate of each fragment is produced by the linear interpolation of the texture homogeneous coordinates of *p*_1_, *p*_2_, *p*_3_, and *p*_4_. To compensate for the perspective distortion effects, the texture coordinate (*x*_*p*_, *y*_*p*_) of each fragment is divided by its 4th component *w_p_* to derive correct coordinate in the fragment shader of GPU. At last, these texture coordinates are used to sample the projection image of this projection view, and the obtained sample values are accumulated into the corresponding voxels of the output texture representing the slice. These calculations finish the backward-projection to the slice from one projection view. Note that the sample positions usually do not coincide with the detector units, the final values of the sample positions are produced by nearest-neighbor interpolation or bilinear interpolation. 

 The above procedure is executed repeatedly until every volume slice is processed from every projection view, thus the entire reconstructed volume is updated.

### 2.2. Utilization of Rotational Symmetry in Projection Layout

**Figure 3:**
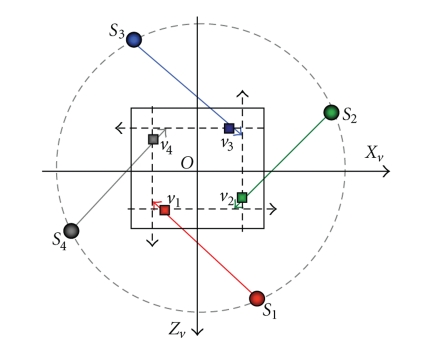
Rotational symmetry in projection layout.

In circular cone-beam volume reconstruction, there are two types of geometric symmetries, which are referred to as the rotational symmetry [2, 7] and vertical symmetry. The rotational symmetry, or 90-degree symmetry, is shown in Figure 3. That is, the pair of the x-ray source *S*_1_ and the voxel position *v*_1_ can be replicated by rotating it across 90°, 180°, and 270° intervals respectively to produce the other three pairs of (*S*_2_, *v*_2_), (*S*_3_, *v*_3_), and (*S*_4_, *v*_4_). This means that they share the same geometric relation in projection layout. The backward-projection can be significantly speeded up by the utilization of rotational symmetry, since the geometry transform matrix described in formula (1) and sample positions in projection images are calculated only once for four symmetric projection views. There is still another kind of rotational symmetry, that is, two pairs of source and pixel positions are symmetric with respect to a diagonal line, which is also called complement symmetry [2]. Constrained by the inherent parallelism offered by the four color channels of GPU, we only utilize the 90-degree symmetry to accelerate back-projection by packing four rotational symmetric projections into four color channels. Our GPU-based backward-projection algorithm using the 90-degree symmetry is as following.

**Step 1**

Arrange the projection images in the four rotational symmetric views of *θ*, *θ* + 90°, *θ* + 180°, and *θ* + 270° as one group, and pack them into the four color channels (red/green/blue/alpha or RGBA) of a 2D-texture *ProjTex*, one projection image per channel.

**Step 2**

Employ four textures *SliceTex1*, *SliceTex2*, *SliceTex3* and *SliceTex4* to save the backward-projected values for four slices, respectively. Each of the four textures has *individual* four color channels, and each channel is used to save the backward-projected values from one projection view.

**Step 3**

In GPU vertex shader, the computation described in formula (1) is only done once from projection view *θ* for each slice by using the algorithm presented at Section 2.1, to produce the projection texture coordinates that are identical for the four symmetric projection images.

**Step 4**

In GPU fragment shader, the projection images in the four symmetric views are backward-projected and accumulated to the four slice textures, respectively, according to the projection texture coordinates produced by the vertex shader and the succeeding rasterizer of GPU. The four slice textures are then rendered to GPU frame buffer in the same pass by the Multiple Render Targets (MRT) technique of OpenGL.

**Step 5**

The above procedures are repeated with Ping-Pong technique, until the projection images of all views are backward-projected and accumulated to the four slices. The effort of backward-projection from full 360° arc is reduced to one 90° arc by using the rotational symmetry.

**Step 6**

**Figure 4:**
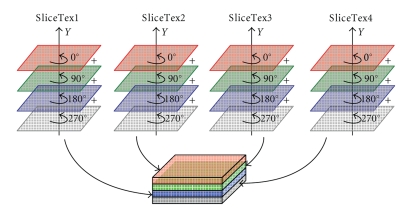
Strategies for slices accumulating and packing.

A new rendering pass is appended in the end. In this pass, as shown in Figure 4, the texture coordinates in the *G*, *B* and *A* channels of the four slice textures are rotated by 90°,180°, and 270°, respectively, and the data in the RGBA channels of each slice texture are respectively accumulated and packed into an output texture with four channels, one slice per channel, which is then downloaded to system memory. Now the four slices have been updated by the projection data from all the views on circular trajectory. The method increases the speed of downloading data by taking advantage of the 4-channel RGBA parallelism, and avoids the calculation of slices accumulating in CPU.

**Step 7**

The above processes are repeated for every volume slice from every projection view, and then the entire reconstructed volume is updated.

### 2.3. Utilization of Vertical Symmetry in Circular Cone-Beam Projection Layout

**Figure 5:**
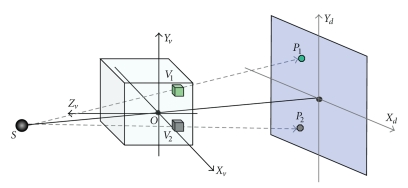
The vertical symmetry in circular cone-beam projection layout.

Another type of symmetry is known as vertical symmetry in circular cone-beam projection layout. As shown in Figure 5, the vertices *v*_1_ and *v*_2_ of the reconstructed volume are vertical symmetric with respect to the central scanning plane (*Z*_*v*_, *X*_*v*_), that means when the coordinate of *v*_1_ is (*x*_*v*_, *y*_*v*_, *z*_*v*_), the coordinate of *v*_2_ is (*x*_*v*_, −*y*_*v*_, *z*_*v*_). According to formula (1), if the projection coordinate of *v*_1_ in detector space is (*x*_*d*_, *y*_*d*_), then that of *v*_2_ is (*x*_*d*_, −*y*_*d*_) in the circular cone-beam scanning case. That is, their *X*_*d*_ coordinates stay the same, while their *Y*_*d*_ coordinates are opposite. 

 We use the property of vertical symmetry to decrease the amount of backward-projection positions calculation. When loading a projection image into GPU memory, we read the data in the upper half of the projection image along *Y*_*d*_ axis, but read the data in the lower half in the opposite order of *Y*_*d*_ axis, that is, fold the projection image. Then we pack the two halves of the projection image into two color channels of a 2D-texture, respectively. Thus the projection positions of the vertices of two vertical symmetrical slices in the projection image are identical, consequently only half of projection positions are needed to calculate for backward-projecting a projection image to the reconstructed volume.

## 3. Support for Large Data Volume

**Figure 6:**
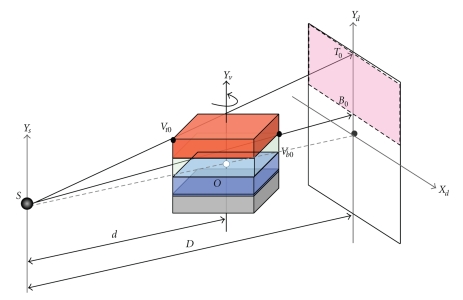
The partitioning scheme of the reconstructed volume and projections.

As for GPU-accelerated algorithms, the projection data should be firstly loaded into graphic card memory so as to be called by GPU, which required expensive data transfers between graphic card memory and system memory due to bandwidth limit. Since the reconstruction of each slice needs the projection images from all projection views, we try to load all the projection images into graphics card memory at one time for saving data transfer time. Currently, graphics cards have typically 512 MB or 768 MB of RAM. If the amount of projection data exceeds the graphic card memory capacity, the projection data have to be partitioned into blocks to fit into the graphic card. A new partitioning scheme is employed in our program. As shown in Figure 6, the reconstructed volume is divided into several chunks, each of which is a stack of the slices of volume. The projection data for reconstructing a chunk do not require complete full sized projection images, but only the blocks contained in a rectangular shape. The height of the rectangle is greatest when the diagonal of volume slices is perpendicular to the detector plane. Considering these properties, we divide projection images into the same number of blocks as volume chunks. The size of each block may be calculated by the following formulas (2)–(4).

Let *d* be source-to-rotation center distance, *D* be source-to-detector distance, and *H_D_* be detector height, then we can get the maximum height of the reconstructed volume:

(2) $$H_{V}=\frac{d\;\cdot \;H_{D}}{D+\left(\sqrt{2}/2\right)H_{D}}.$$

If the reconstruction volume is divided into *N* chunks, then the height of each chunk is *H_V_*/*N*, and the top coordinate and bottom coordinate of the *n*th chunk along the *Y*_*v*_ axis can be calculated according to the formula (3):

(3) $$\begin{array}{cc} V_{tn} & =H_{V}\left(\frac{1}{2}-\frac{n}{N}\right)\\ V_{bn} & =H_{V}\left(\frac{1}{2}-\frac{n+1}{N}\right) \end{array}\;n=0,1,\ldots ,N-1.$$

The corresponding top projection position *T_n_* and the bottom projection position *B_n_* of the *n*th chunk along *Y*_*d*_ axis in detector may then be calculated by formula (4), as shown in Figure 6:

(4) $$\begin{array}{ll} & T_{n}=\frac{D\;\cdot \;V_{tn}}{d-\left(\sqrt{2}/2\right)H_{V}}\\ & B_{n}=\frac{D\;\cdot \;V_{bn}}{d+\left(\sqrt{2}/2\right)H_{V}} \end{array}\;n=0,1,\ldots ,N-1.$$

According to the obtained parameters *T_n_* and *B_n_*, we can know how to divide each projection image into blocks. Only one related projection data block is uploaded into graphics card memory each time for reconstructing one chunk, and backward-projected to all slices of the chunk. The actual number of partitions in the program will depend on the size of projection data and the size of graphic memory. Different from the partitioning scheme simply introduced in [8], our method decomposes projections into blocks by utilizing vertical symmetry and rotational symmetry, and reconstructs the volume slices at the vertical symmetrical positions in the meanwhile. The reconstructed volume slices are assembled in the end. The partitioning scheme can avoid repeated data transfer and speed up volume reconstruction. 

 In conjunction with the geometric symmetries presented in Sections 2.2  and 2.3, the cone-beam CT image reconstruction method for large data volume is summarized as follows.

**Step 1**

The projection at each view is divided into blocks according to our partitioning scheme, and each block is further decomposed into two vertical symmetric subblocks for utilizing vertical symmetry, that is, an upper sub-block and a lower sub-block.

**Step 2**

The data in the upper sub-block of current block are packed into a texture with RGBA color channels every four rotational symmetric views, and the data in the lower sub-block are also packed into another texture with RGBA color channels every four rotational symmetric views. All data in the current block are transferred into graphic card memory from system memory by these textures for subsequent image reconstruction.

**Step 3**

According to the algorithms presented in Sections 2.2  and 2.3, four vertical symmetric slices are reconstructed in parallel each time using the current projection data block from four rotational symmetric views. The process is repeated until all slices in current data chunk are reconstructed.

**Step 4**

Once the image reconstruction for every four slices is achieved, they are packed into an output texture with four channels, one slice per channel, and downloaded into system memory.

**Step 5**

The above 2nd to 4th steps are executed repeatedly until every volume slice in every chunk is processed from all projection views, then the image reconstruction for entire volume is achieved.

## 4. Numerical Experiments

To test the gain of our GPU-based acceleration scheme, we have used the FDK algorithm that applies the GPU-based backward-projection to reconstruct images from computer simulated data and real mouse data acquired with a microcone-beam CT system. The PC used has a 1.83 GHz Intel Xeon 5120 dual-core CPU with 8 GB of system memory. The graphics card is NVIDIA Qurdro FX4600 model with 768 MB of memory. For the simulated data, the source-to-rotation center (SOD) is set to 1660.0 mm, the source-to-detector distance (SDD) is 1900.0 mm, and the size of each detector bin is 0.127 mm × 0.127 mm. These parameters are set according to a real industry CT system in our laboratory. The micro-CT source-to-detector distance is 570.0 mm and source-to-rotation center is 390.0 mm. Its detector bin is 0.049 mm × 0.049 mm. 

**Figure 7:**
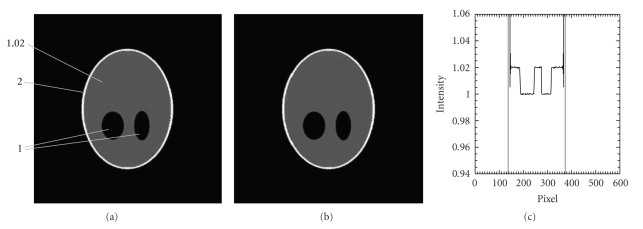
Shepp-Logan phantom reconstruction (middle slice): (a) is the true image, and (v) is the reconstruction by use of GPU-accelerated FDK program, (c) is a line profile along *y* = 200 pixel. The dotted line corresponds to GPU reconstructed image profile and the solid line corresponds to the true phantom profile. Little difference is shown in the line profiles.

 We have performed reconstructions for Shepp-Logan phantom volumes with 512^3^ and 1024^3^ voxels by use of the FDK algorithm with a GPU-based backward-projection. In this reconstruction, the detector array sizes are 512^2^ and 1024^2^, and the numbers of projection views are 360 and 720, respectively. The programmable pipeline of FX4600 GPU supports 32-bit float precision calculation*,* and our GPU-based reconstructions show the equivalent image quality as our CPU-based implementations, as shown in Figure 7.

Since our graphics card memory is 768 MB, the projections for reconstructing the volume with 512^3^ voxels can be uploaded into graphics card memory at one time, and the backward-projection takes 7.2–7.7 seconds. While the projections in 32-bit float precision for reconstructing the volume with 1024^3^ voxels are too large to be transferred to graphics card memory at one time. The projections need to be partitioned to fit into the graphics card memory on the basis of our partitioning scheme presented in Section 3. We decompose the reconstruction volume into 4 chunks, and correspondingly the projection data are also divided into 4 blocks. Since the height of each detector unit is 0.127 mm, the height of the detector *H_D_* is 1024 × 0.127 mm = 130.038 mm. According to formulas (2)–(4), we can know the maximal size of reconstruction volume *H*_*V*_ = 108.368 mm, and the top projection coordinates and bottom projection coordinates of these 4 chunks are: *T*_0_ = 65.024 mm, *B*_0_ = 29.643 mm; *T*_1_ = 32.512 mm, *B*_1_ = 0; *T*_2_ = 0, *B*_2_ = −32.512 mm; *T*_3_ = −29.643 mm, *B*_3_ = −65.024 mm, respectively. That is, about 279 rows of projection data at each view are needed for reconstructing the 0th chunk of volume, 256 rows for the 1th chunk, also 256 rows for the 2th chunk, and 279 rows for the 3th chunk. Altogether (279 + 256 + 256 + 279)/1024 ≈ 1.045 times projection data are needed to transfer from system memory to graphics card memory for reconstructing the whole volume. Hence, as compared to the methods presented in papers [5, 9], the amount of transferred data is greatly reduced by our division scheme, and the backward-projection time is only 101.9–104.8 seconds. 

**Figure 8:**
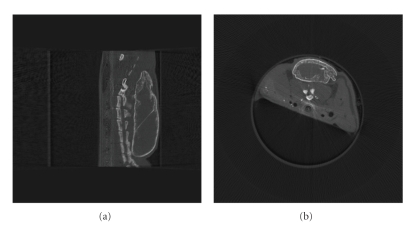
A mouse reconstructed by use of GPU-accelerated FDK program from the micro-CT data: (a) the sagittal slice of the mouse, (b) the middle transverse slice of the mouse.

 We have also applied the GPU-accelerated FDK algorithm to the real mouse data acquired with a microcone-beam CT scanner. The projection size for each projection view is 1600 × 980, and data were collected at a total number of 360 views. The reconstructed image array is 512^3^. Again, this system is too large for one shot reconstruction, and the projection data needs to be partitioned. The backward-projection time is about 14.5–15.2 seconds given by 3 partitions of the projection data. Figure 8 shows the sagittal slice and the middle transverse slice of the reconstruction image. As compared to our CPU-based implementation of the FDK algorithm on the same computer, the backward-projection time is reduced by about a factor of 110–120 without compromising image quality.

## 5. Conclusion

In the work, we have investigated and implemented a GPU-based 3D cone-beam CT image reconstruction algorithm for large data volume, and evaluated the GPU-based implementations by use of computer-simulation data and real micro-CT data. The GPU-based implementation using geometric symmetries has speeded up the backward-projection process by about 110–120 times for a circular cone-beam scan, as compared to the CPU-based implementation on the same PC. The volumes reconstructed by GPU and CPU have virtually identical image quality. Further work is in progress to apply our algorithms to the iterative image reconstruction methods of cone-beam CT.
